# Dynamic switch of immunity and antitumor effects of metformin in rat spontaneous esophageal carcinogenesis

**DOI:** 10.1007/s00262-021-03027-x

**Published:** 2021-08-16

**Authors:** Ryohei Takei, Tomoharu Miyashita, Satoshi Takada, Hidehiro Tajima, Itasu Ninomiya, Hiroyuki Takamura, Sachio Fushida, Ai Harashima, Seiichi Munesue, Shintaro Yagi, Noriyuki Inaki, Tetsuo Ohta, Yasuhiko Yamamoto

**Affiliations:** 1 Department of Gastroenterologic Surgery, Kanazawa University Graduate School of Medical Sciences, Kanazawa, 920-8640 Japan; 2 Department of Surgical Oncology, Kanazawa Medical University Hospital, 13-1 Takaramachi, Kanazawa, 920-8640 Japan; 3 Department of Biochemistry and Molecular Vascular Biology, Kanazawa University Graduate School of Medical Sciences, Kanazawa, 920-8640 Japan

**Keywords:** Gastroduodenal reflux esophagitis, Esophageal cancer, Chronic inflammation, Tumor microenvironment, Metformin

## Abstract

Chronic inflammation contributes to tumor development by creating a local microenvironment that facilitates neoplastic transformation and potentiates the progression of cancer. Esophageal cancer (EC) is an inflammation-associated malignancy with a poor prognosis. The nature of the switch between chronic inflammation of the esophagus and EC-related immunological changes remains unclear. Here, we examined the dynamic alterations of immune cells at different stages of chronic esophagitis, Barrett’s esophagus (BE) and EC using an esophageal spontaneous carcinogenesis rat model. We also investigated the anticancer effects of metformin. To stimulate EC carcinogenesis, chronic gastroduodenal reflux esophagitis via esophagojejunostomy was induced in 120 rats in metformin-treated and non-treated (control) groups. After 40 weeks, BE and EC developed in 96.7% and 63.3% of the control group, and in 66.7% and 23.3% of the metformin-treated group, respectively. Flow cytometric analysis demonstrated that the balance of M1/M2-polarized or phospho-Stat3-positive macrophages, regulatory T, cytotoxic T, natural killer (NK), NK T cells, and Th17 T cells was dynamically changed at each stage of the disease and were resolved by metformin treatment. These findings clarify the immunity in esophageal carcinogenesis and suggest that metformin could suppress this disease by improving the immunosuppressive tumor microenvironment and immune evasion.

## Introduction

Esophageal cancer (EC) is an aggressive cancer with a poor prognosis. More than 90% of EC cases are histologically comprised of esophageal squamous cell carcinoma (ESCC). The incidence of esophageal adenocarcinoma (EAC) has recently increased dramatically in the USA and several Western countries [1].

EAC generally originates from Barrett’s esophagus (BE) and is pathologically characterized by intestinal metaplasia of the distal esophagus, followed by gastroesophageal reflux disease (GERD) and subsequent chronic esophagitis. Chronic esophagitis was reported to result in a 4.5- and 29.8-fold increase in the relative risk of developing EAC and BE, respectively [2].

Carcinogenesis is caused by chronic inflammation and the production of reactive oxygen species, leading to oncogenic mutations [3]. Previous studies have reported somatic mutations in cancer-related genes in EAC, such as *TP53* [4], *TP16* [5], *SMAD4* [6], *PIK3CA* [7], *EGFR* [8], and *APC* [9]. Additionally, chronic inflammation forms a cancer microenvironment that can induce the proliferation of cancer cells and enhance their survival. Therefore, inflammatory responses in cancer are a two-edged sword, with tumor-suppression (cancer-killing) and tumor-promoting dynamic changes in immune cell populations. The tumor-suppressive and cancer-killing cell types include natural killer (NK) cells, NK T (NKT) cells, CD8^+^ T lymphocytes, and M1-polarized macrophages (M1Ms). In contrast, regulatory T cells (Tregs) and M2-polarized macrophages (M2Ms) are anti-inflammatory cell types that promote tumor formation in the cancer microenvironment. Inflammation can also activate intracellular signaling proteins, such as nuclear factor-kappa B (NF-κB) and signal transducer and activator of transcription 3 (STAT3) via inflammation mediators that include interleukin-6 (Il-6). Activated STAT3 plays an important role in tumor growth and survival [10], and in the suppression of antitumor immunity by enhancing the activity of Tregs and M2Ms [11, 12].

Our Gastroenterologic Surgery group previously established an esophageal spontaneous carcinogenesis rat model, which shows symptoms similar to those of human EC, by inducing gastroduodenal reflux esophagitis after performing an esophagojejunostomy [13]. This rat model allowed us to examine changes in the composition of esophageal immune cells at all stages of esophageal carcinogenesis, including proliferative hyperplasia (PHP), BE, ESCC, and EAC, to clarify the mechanisms underlying carcinogenesis stimulation in EAC through the inflammatory reaction.

Metformin is a drug used to treat type 2 diabetes. The drug also has anti-hyperglycemic and unexpected anticancer effects. Epidemiological studies have associated metformin with a reduction in the incidence and mortality rates of many solid tumors [14]. Clinical evaluation of the chemopreventive and anti-neoplastic effects of metformin is ongoing in phase III trials for several cancers, including endometrial cancer, colorectal cancer, and breast cancer [15]. The anticancer mechanisms of metformin include the activation of AMP kinase (AMPK), which can preferentially kill cancer-initiating/stem cells of glioblastoma, breast, and ovarian cancers [16–18]. Furthermore, the inactivation of STAT3 by metformin reportedly inhibits cell growth and induces apoptosis in esophageal, pancreatic, and breast cancers [19–21].

The aims of this study were to elucidate the dynamic changes in immune cell populations in the esophagus during the carcinogenic transition from GERD to Barrett’s metaplasia and finally to EC. Additionally, the preventive effects of metformin on esophageal carcinogenesis were investigated by evaluating the esophageal microenvironment using our rat surgical model of duodenogastroesophageal reflux (DGER).

## Materials and methods

### Experimental animals and metformin treatment

Sprague–Dawley male rats (4 weeks old) weighing ~ 250 g were used for all experiments. The animals were housed at a constant temperature of 22 ± 3 °C and 55 ± 5% humidity under a 12-h light–dark cycle. The rats were fed standard solid chow (CRF-1; Charles River Laboratories, Yokohama, Japan) and tap water. Following a 24-h fast, an upper abdominal incision and esophagojejunostomy were made under isoflurane inhalation anesthesia to induce DGER. The animals were divided into three groups. The sham-operated control group (*n* = 5) underwent only laparotomy and the rats were fed standard chow. The control group (*n* = 60) underwent esophagojejunostomy and were fed standard chow (CRF-1). The metformin-treated group (*n* = 60) underwent esophagojejunostomy and were fed CRF-1 containing 1.3% metformin (900 mg/kg/day). The dosage of metformin was based on a non-clinical study of the drug toxicity performed by Sumitomo Dainippon Pharma Corporation (Osaka, Japan). Metformin was provided by Sumitomo Dainippon Pharma Corporation and Towa Pharmaceutical Corporation (Osaka, Japan). At 10 (*n* = 10), 20 (*n* = 10), 30 (*n* = 10), and 40 (*n* = 30) weeks after the operation, the rats were subjected to laparotomy and thoracotomy under isoflurane inhalation for the extraction of specimens. After placing a 24G needle into the left ventricle and making a small incision in the right auricle, whole perfusion was performed with normal saline. After the animals were sacrificed by deep inhalation anesthesia, the entire esophagus and anastomosed jejunum were extracted for histopathological and flow cytometry analyses. This study was approved by the Institutional Animal Care and Use Committee of the Graduate School of Medical Sciences, Kanazawa University (AP-153540; Kanazawa, Japan).

### Pathological assessment

Extracted tissues were fixed in 10% neutral-buffered formaldehyde for at least 24 h. After fixation, the specimens were cut at 3-mm intervals along the long axis and embedded in paraffin. Each paraffin block was sliced into 5-µm sections and stained with hematoxylin and eosin. Histological findings in the specimens were classified into the following three categories according to a previous report [23]:

1. Squamous proliferative hyperplasia (PHP), which features thickening of the epithelium to twice that of a normal epithelium with acanthosis, elongation of the papillae, and parakeratosis. Other features include thickening of the basal layer of the squamous epithelium and preservation of a stratified appearance.
2. Barrett’s metaplasia (BM), which features replacement of the esophageal squamous epithelium with columnar-lined epithelium comprised of gastric and/or intestinal cells.
3. Carcinoma, featuring cellular, and structural atypism with epithelial invasion into the submucosal layer. EAC shows dysplastic glandular cell growth with both atypia and invasiveness. ESCC is a type of squamous cell dysplasia with marked cellular and structural atypism.

For immunohistochemical studies, the sections were deparaffinized, rehydrated, and then treated with citrate buffer (pH 6.7) at 95 °C for 20 min. Following endogenous peroxidase blocking with 3% hydrogen peroxide, the sections were incubated overnight at 4 °C with specific antibody to pStat3 (1:100; anti-Stat3 [phospho S727], ab30647; Abcam, Cambridge, UK). The slides were then incubated with horseradish peroxidase (HRP)-labeled anti-rabbit or anti-mouse IgG (Dako, Foster City, CA, USA) for 1 h. Immunostaining was visualized by incubation with 3,3-diaminobenzidine tetrahydrochloride (DAB; Dako) for 1–2 min and counterstained with Meyer hematoxylin for 30 s. Negative controls were prepared by replacing the primary antibodies with buffer solution.

### Isolation of immune cells

The proximal or distal esophagus of anastomosed rats was dissected and flushed with Dulbecco’s modified Eagle’s medium (DMEM). The tissue was minced with scissors and placed in a 50 mL tube with 10 mL collagenase solution (DMEM supplemented with 1 µg/mL type 2 collagenase and 1 µg/mL DNase I; Sigma-Aldrich, St. Louis, MO, USA). Following incubation for 30 min at 37 °C with gentle shaking, 10 mL DMEM containing 10% fetal bovine serum (FBS) was added to the collagenase solution. Isolated cells were strained through a 40 μm filter and washed twice with fluorescence-activated cell sorting (FACS) buffer (PBS containing 2% FBS). Cells were then resuspended in 600 µL FACS buffer for analysis.

### Flow cytometry analyses

After treatment with Fc block (anti-rat CD16/CD32 antibody; BD Biosciences, San Jose, CA, USA), the cells were incubated with antibodies specific to fluorescein isothiocyanate (FITC)-conjugated anti-rat CD3, phycoerythrin (PE)-Cy7-conjugated anti-rat CD4, PE-conjugated anti-rat CD8a, adenomatous polyposis coli (APC)-conjugated anti-rat NK1.1 (BioLegend, San Diego, CA, USA), PE-conjugated anti-rat CD25 (eBioscience, Waltham, MA, USA), PE-Cy7-conjugated anti-rat CD11b/c (BD Bioscience), FITC-conjugated anti-rat CD68 (AbD Serotec, Raleigh, NC, USA), PE-conjugated anti-rat CD86 (BioLegend), and APC-conjugated anti-rat CD163 (AbD Serotec). Dead cells were excluded from flow cytometry analysis by adding propidium iodine solution. Cell numbers were evaluated using the BD Cell Viability kit (BD Biosciences). For the detection of Tregs, intracellular FoxP3 staining was performed using an anti-rat FoxP3-APC staining kit (eBioscience) according to the manufacturer’s instructions. To detect intracellular cytokines, 1 h brefeldin A (BD Biosciences) treatment and surface marker staining were performed, followed by fixation and permeabilization with the Cytofix/CytoPerm kit (BD Biosciences). Samples were then stained with specific antibodies for eFlour660-conjugated anti-rat tumor necrosis factor-alpha (TNF-α; eBioscience), Alexa647-conjugated anti-rat interferon-gamma (IFN-γ; BioLegend), Alexa647-conjugated anti-rat IL-10 (BD Biosciences), Alexa405-conjugated anti-rat transforming growth factor-beta (TGF-β; Novus Biologicals, Centennial, CO, USA), PE-conjugated anti-rat IL-17 (Life Technologies, Carlsbad, CA, USA), and Alexa647-conjugated anti-rat Stat3 (pTyr705 and pSer727; BD Biosciences). Flow cytometry analysis was performed using FACSAria Fusion (BD Bioscience). The obtained data were re-analyzed using FlowJo v 9.6.1 software (FlowJo, Ashland, OR, USA). Unstained, single stains, and fluorescence minus one (FMO) controls were used to set compensation and gates.

## Statistical analyses

Statistical analyses were performed using Student’s *t*-test or ANOVA with the Tukey–Kramer post-hoc test using Ekuseru-Toukei 2015 software (Social Survey Research Information Co., Ltd., Japan). *P* < 0.05 was considered statistically significant.

## Results

### Chronological evaluation of changes in immune cell characteristics during inflammation and carcinogenesis

**Fig. 1:**
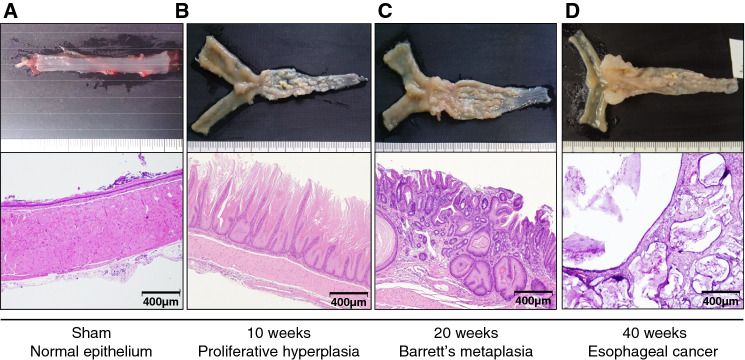
**a** Sham-operated control rats (Sham). Normal epithelium. **b** A distal esophagus at 10 weeks after the operation (10 weeks). The esophagus was macroscopically widened and thickened by chronic gastroduodenal content reflux esophagitis, squamous proliferative hyperplasia (PHP) and slight thickening of the lamina propria were microscopically observed. Magnification, × 10. **c** A distal esophagus at 20 weeks after the operation (20 weeks). Intestinal metaplasia-like lesions were observed without a sharp demarcation between esophagus and jejunum. Pathologically, the hyperplastic changes became severer and Barrett’s metaplasia were evident in some rats. Magnification, × 10. **d** A distal esophagus at 40 weeks after the operation (40 weeks). Overt esophageal cancer was seen as an ulcer or a mass at 30 to 40 weeks. Pathologically, esophageal adenocarcinoma (EAC) and esophageal squamous cell carcinoma were observed. Magnification, × 10

**Fig. 2:**
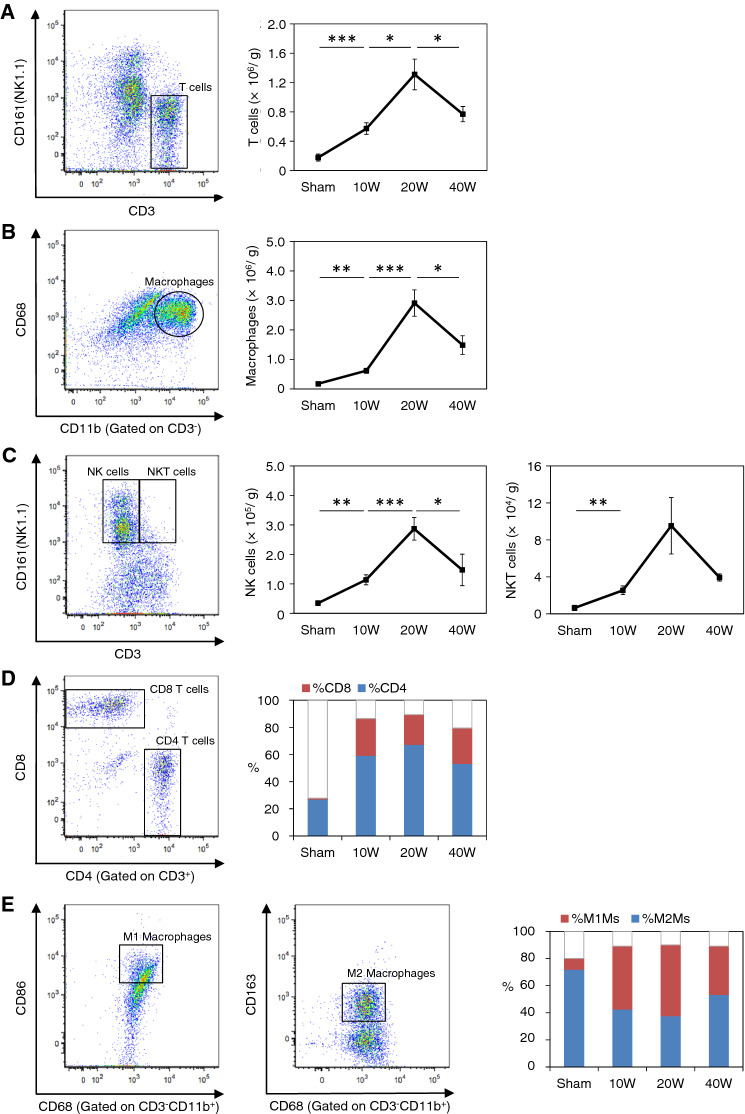
**a–c** Flow cytometric analyses of the number of CD3^+^ T lymphocytes, pan macrophages (CD3^−^CD11b^+^CD68^+^), NK cells (CD3^−^NK1.1^+^), and NKT cells (CD3^+^NK1.1^+^) in sham-operated control rats (Sham) and in rats at 10, 20, and 40 weeks after the surgery (10 W, 20 W and, 40 W, respectively). **d** The proportions of CD4^+^ and CD8^+^ T cells in CD3^+^ T cells. **e** The proportions of CD11b^+^CD68^+^CD86^+^ M1-like (M1Ms) and CD11b^+^CD68^+^CD163^+^ M2-like (M2Ms) macrophages in pan macrophages. Data are presented as the mean ± SEM; n = 6 per group; *, *p* < 0.05; **, *p* < 0.01; ***, *p* < 0.001

In the esophageal spontaneous carcinogenesis model, esophagitis spreads macroscopically from the anastomotic region to the oral side of the esophagus with irregular wall thickness and dilatation over time (Fig. 1). Intestinal metaplasia-like lesions were observed without a clear demarcation between the esophagus and jejunum 20 weeks after esophagojejunostomy (Fig. 1c, upper panel). Overt EC was observed as an ulcer or mass ~ 40 weeks post-surgery (wps) (Fig. 1d, upper panel). Histologically, squamous PHP and slight thickening of the lamina propria were observed in all the animals at 10 wps (Fig. 1b, lower panel). These hyperplastic changes became more severe. BM was evident in some rats at 20 wps and EAC and ESCC were observed at ~ 40 wps (Fig. 1c, d). The infiltration of immune cells, mostly small and round cells, such as lymphocytes, into the epithelium and lamina propria of the esophagus was observed in the early stage at 10 wps (Fig. 1b). Infiltration became more severe at 20 wps (Fig. 1c). Quantitative flow cytometry revealed markedly increased CD3^+^ T lymphocytes and CD11b^+^CD68^+^ macrophage numbers in esophageal lesions (Fig. 2a, b). Increases of 7.4- and 16.5-fold, respectively, were evident compared to those in the sham-operated control group at 20 weeks (Fig. 2a, b). Thereafter, the infiltration of these cells declined (Fig. 2a, b). A similar pattern of cell infiltration was also observed in NK and NKT cells (Fig. 2c). In terms of the content of CD3^+^ T lymphocytes, the percentage of CD4^+^ T cells increased from 27% (sham-control group) to 67% (control group) (Fig. 2d). Surprisingly, the percentage of CD8^+^ cells increased from 0.7% (sham-control group) to ~ 27% (control group) during the observation period (Fig. 2d). CD11b^+^CD68^+^CD163^+^ M2M macrophages were more predominant in sham-operated esophagus samples (Fig. 2e). However, the CD11b^+^CD68^+^CD163^−^ M1M macrophage ratio increased after surgery, peaked at 20 wps, and decreased thereafter (Fig. 2e).

### Metformin treatment inhibits development of BE and EC

**Fig. 3:**
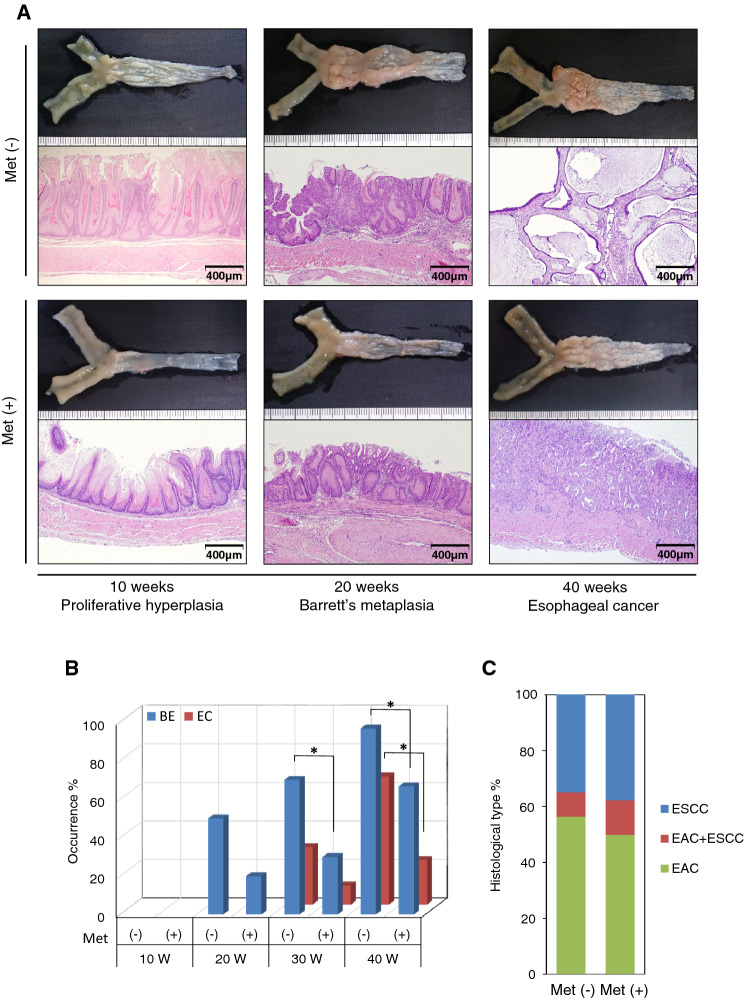
**a** Macroscopic and microscopic findings of the distal esophagus in non-treated and metformin-treated group at 10, 20, and 40 weeks after the esophagojejunostomy. Wall thickening and dilatation changes of the esophagus as well as the lesion sizes of Barrett’s metaplasia and esophageal cancer were attenuated in the metformin-treated group [Met ( +)] compared with those in the non-treated control [Met (-)]. Microscopic findings (H&E stain; magnification, × 10). **b** The incidence rate of Barrett’s metaplasia and esophageal cancer in rats with or without metformin treatment [Met ( +) or Met (-), respectively] at 10 (10 W, n = 10), 20 (20 W, *n* = 10), 30 (30 W, *n* = 10), and 40 weeks (40 W, *n* = 30) after the esophagojejunostomy. *, *p* < 0.05. **c** The proportion of histological types of esophageal cancer in rats with or without the metformin treatment [Met ( +) or Met (-), respectively]. ESCC, esophageal squamous cell carcinoma; EAC, esophageal adenocarcinoma

We next investigated the effects of metformin using this animal model. Compared to untreated controls, metformin treatment markedly attenuated wall thickening and changes in the dilation of the esophagus (Fig. 3a). The frequencies and sizes of BE and EC lesions were substantially attenuated after metformin treatment (Fig. 3a, b). Microscopic observations revealed the beneficial effects on esophagitis and the development of BE and EC lesions in the metformin-treated group compared to those in the untreated control (Fig. 3a). Quantitative analyses demonstrated that BE was present in 0, ~ 50, ~ 70, and 96.7% of subjects, and EC was observed in 0, 0, ~ 30, and 66.7% of subjects at 10, 20, 30, and 40 wps, respectively, in non-treated controls (Fig. 3b). In contrast, the metformin-treated group showed a lower incidence of BE (0, ~ 20, ~ 30, and 66.7%) and EC (0, 0, ~ 10, and 23.3%) at 10, 20, 30, and 40 wps, respectively (Fig. 3b). However, we did not observe any differences in the incidence of EAC and ESCC between the metformin-treated and untreated control groups at 40 wps (Fig. 3c). Accordingly, we tentatively defined the “inflammatory phase” as 10 wps, “Barrett’s metaplasia rising phase” as 20 wps, and “carcinogenesis phase” as 40 wps.

### Dynamic alterations in immune cell characteristics during inflammation and carcinogenesis, and response to metformin

**Fig. 4:**
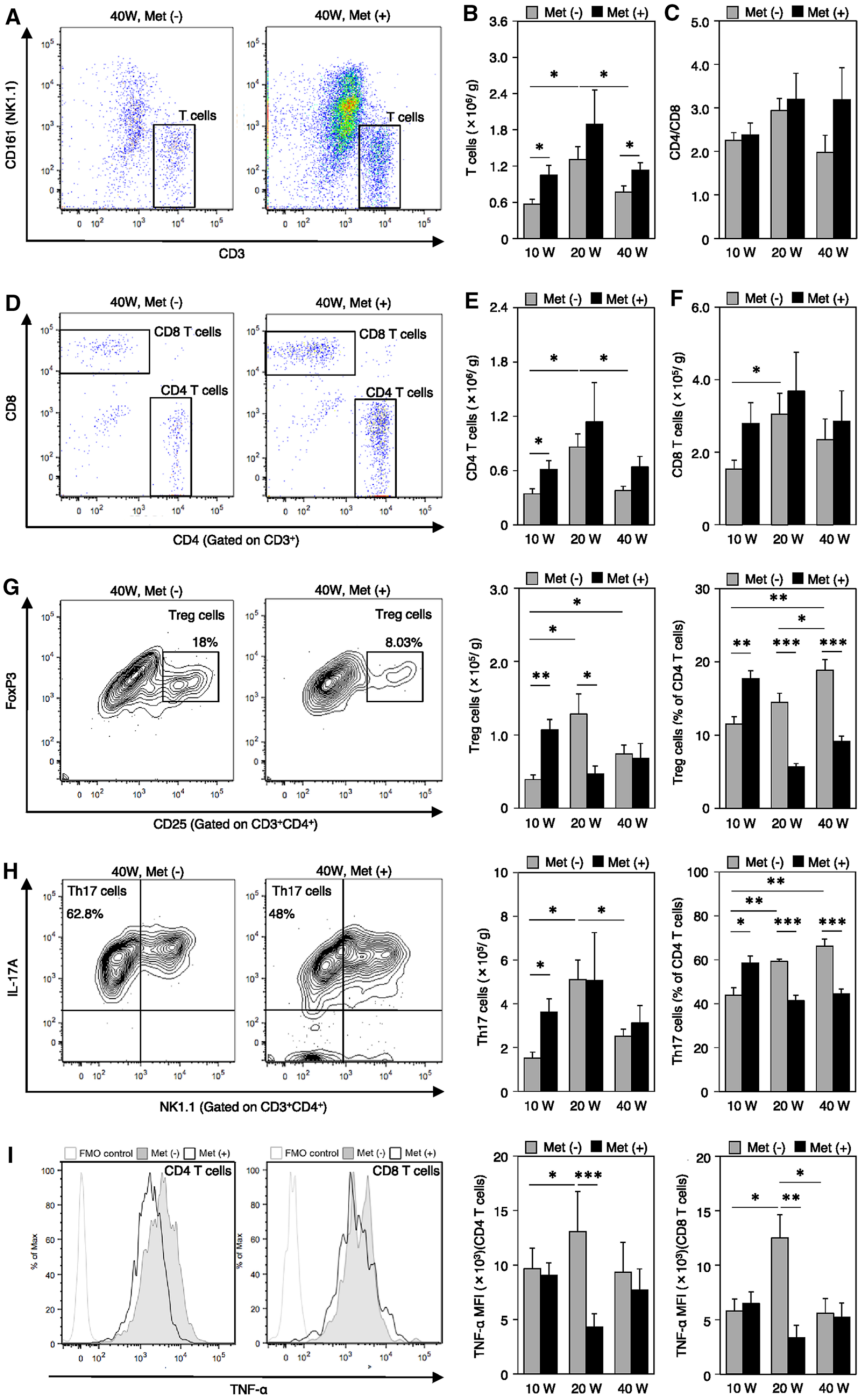

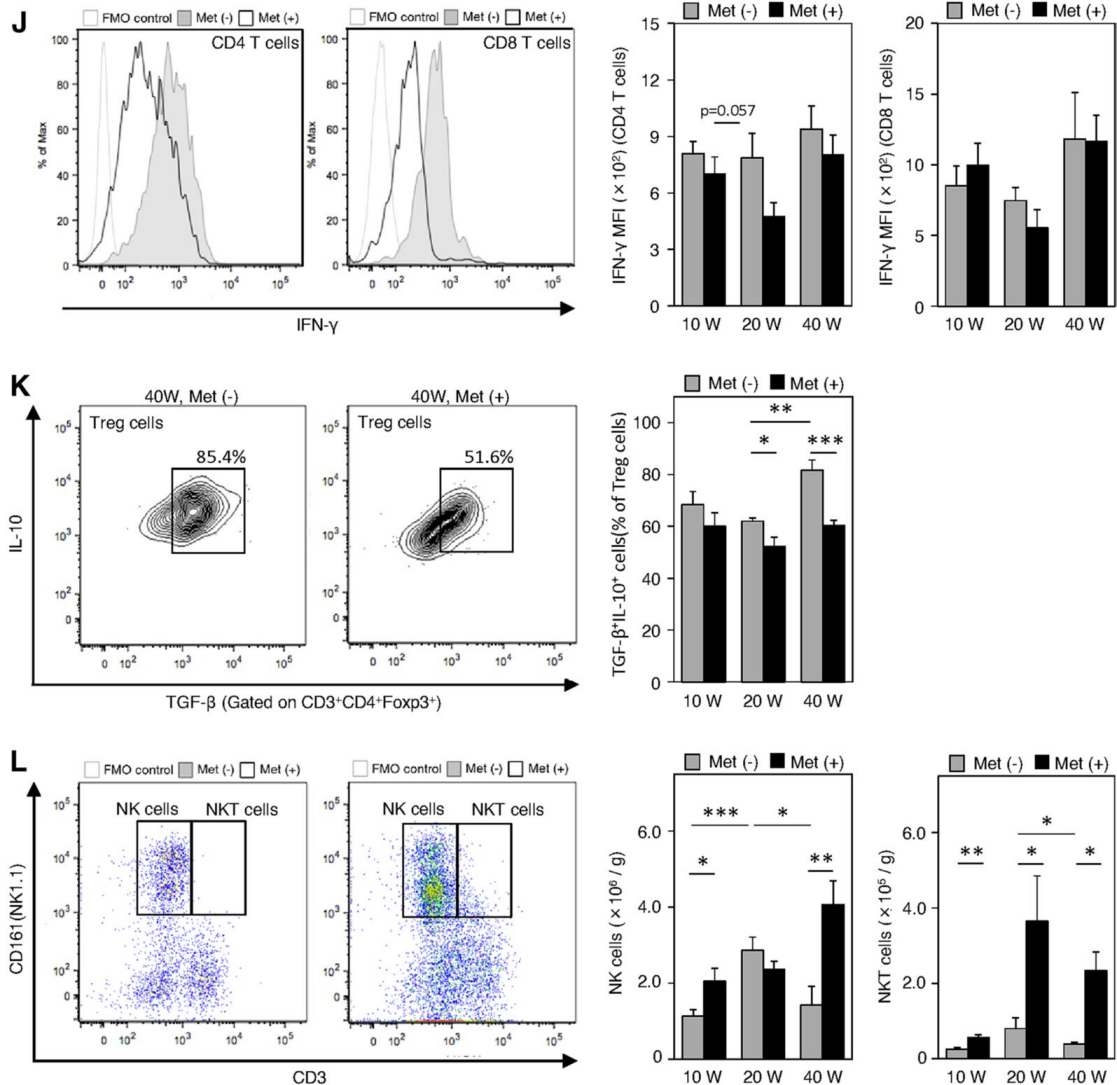
Flow cytometric analyses of characteristics and the number of infiltrating immune cells in the lesions of the esophagus at 10, 20, and 40 weeks (10 W, 20 W, and 40 W, respectively) after the surgery in metformin-treated groups [Met ( +), dark shading] and the non-treated controls (Met (-), gray shading). **a, b** CD3^+^ T cells. **c–f** CD4^+^ and CD8^+^ T cells. **g** Treg cells. **h** Th17 cells. **i, j** TNF-α and IFN-γ expressions in CD4^+^ T cells, and CD8^+^ T cells. MFI, mean fluorescence intensity. **l** NK and NKT cells. **k** TGF-β^+^IL-10^+^ Treg cells in Treg cells. Fluorescence minus one (FMO) controls were used to set gates. Data are presented as mean ± SEM; *n* = 6 per group; *, *p* < 0.05; **, *p* < 0.01; ***, *p* < 0.001

Flow cytometry analyses were performed to characterize infiltrated immune cell populations in esophageal tissues during the inflammatory phase (10 wps), Barrett rising phase (20 wps), and carcinogenesis phase (40 wps) of the metformin-treated group and the non-treated controls. First, we focused on the T cell population (Fig. 4). The population of infiltrated CD3^+^ T cells peaked at 20 wps, but metformin did not affect its infiltration dynamics (Fig. 4a, b). However, metformin treatment significantly increased CD3^+^ T cell numbers at 10 and 40 wps (Fig. 4b). In the inflammatory phase at 10 wps, the numbers of CD4^+^ and CD8^+^ T cells were significantly increased by metformin treatment in the esophagus (Fig. 4d–f). The increased CD4^+^ T cell populations at 10 weeks were CD4^+^CD25^+^Foxp3^+^ Treg cells and CD4^+^NK1.1^−^IL-17A^+^ Th17 cells (Fig. 4g, h). We did not observe any significant changes in the expression of TNF-α and INF-γ in CD4^+^ and CD8^+^ T cells, in the CD4/CD8 ratio, or in the percentage of TGF-β^+^IL-10^+^ Tregs at 10 weeks (Fig. 4c, i–k).

During the transition from the inflammatory phase to the carcinogenesis phase (10 to 40 wps), the percentage of Tregs to total CD4^+^ cells was significantly increased in the non-treated controls (Fig. 4g). In contrast, metformin significantly decreased the Treg cell percentages at 20 and 40 wps (Fig. 4g). Similar changes were also observed in Th17 cells (Fig. 4h). However, the calculated populations of infiltrated Tregs and Th17 cells in the esophagus at 40 wps were not altered by metformin treatment (Fig. 4g, h). These findings suggest that metformin did not markedly affect the regulation of Treg and Th17 cell numbers. However, TGF-β^+^ and IL-10^+^ Tregs were significantly decreased at 40 wps (Fig. 4k).

We next investigated changes in NK and NKT cell populations. NK cells were significantly increased at 10 and 40 wps (inflammatory and carcinogenesis phases, respectively) upon metformin treatment, and the infiltration of NKT cells was significantly increased at all stages in the metformin-treated group compared with that in the non-treated group (Fig. 4l).

**Fig. 5:**
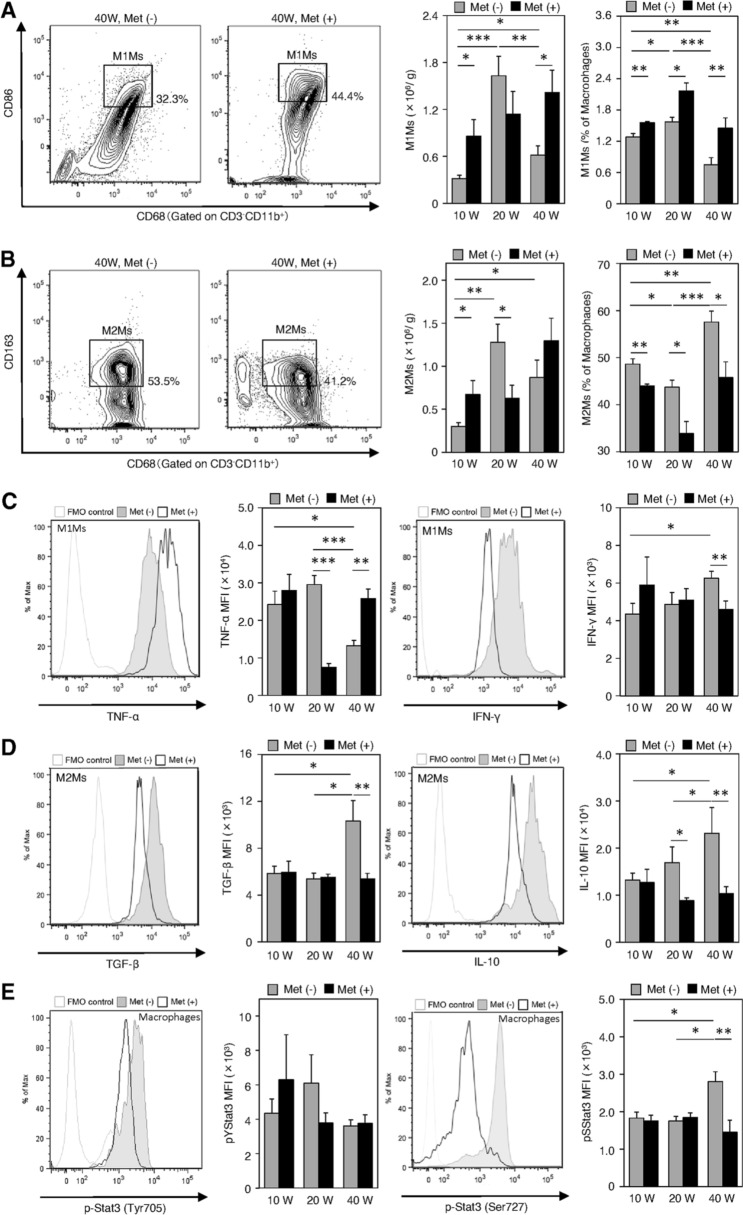
Flow cytometric analyses of characteristics and the number of macrophages infiltrated in the lesions of the esophagus at 10, 20, and 40 weeks (10 W, 20 W, and 40 W, respectively) after the surgery in metformin-treated groups [Met ( +), dark shading] and the non-treated controls (Met (-), gray shading). **a, b** The number and the percentage of the M1-polarized macrophages (M1Ms) and M2-polarized macrophages (M2Ms). **c** Expressions of TNF-α and IFN-γ in M1Ms. MFI, mean fluorescence intensity. **d** Expressions of TGF-β and IL-10 in M2Ms. MFI, mean fluorescence intensity. **e** Levels of phosphorylated Stat3 (p- Stat3) (pTyr705 and pSer727) in macrophages. MFI, mean fluorescence intensity. Fluorescence minus one (FMO) controls were used to set gates. Data are presented as mean ± SEM; *n* = 6 per group; *, *p* < 0.05, **, *p* < 0.01, ***, *p* < 0.001

We next examined changes in the macrophage populations and their characteristics (Fig. 5). The infiltration of CD11b^+^CD68^+^CD86^+^ M1Ms in the esophagus significantly increased at 20 wps relative to that at 10 wps, and then significantly decreased at 40 wps in the non-treated group (Fig. 5a). However, metformin treatment significantly upregulated the number of M1Ms at 10 wps (Fig. 5a). This increase was maintained until 40 wps (Fig. 5a). The percentage of CD11b^+^CD68^+^ CD86^+^ M1Ms relative to total macrophages was ~ 50% until 20 wps, but significantly decreased in the carcinogenesis phase in the non-treated group (Fig. 5a). However, metformin did not decrease the infiltration of M1Ms (Fig. 5a). The calculated number of infiltrated M1Ms significantly increased in the esophagus at 10 and 40 wps in the metformin-treated group compared to those in the non-treated control (Fig. 5a). In contrast, the percentage of CD11b^+^CD68^+^CD163^+^ M2Ms relative to total macrophages was increased at 40 wps (carcinogenesis phase) compared to that at 10 wps in the non-treated group, but was significantly reduced by metformin treatment at all stages, especially at 20 wps (Barrett rising phase) (Fig. 5b). The calculated numbers of M2Ms were increased at 10 wps but significantly decreased at 20 wps in the metformin-treated group compared to the values in the untreated control group (Fig. 5b). These data showed that metformin treatment shifted the M1Ms/M2Ms balance in favor of M1Ms during the observation period. We then examined the changes in the expression of TNF-α and IFN-γ in M1Ms (Fig. 5c), and of TGF-β and IL-10 in M2Ms (Fig. 5d). TNF-α expression was downregulated at 20 wps (Barrett rising phase) in the metformin group (Fig. 5c). This change was also apparent at 40 wps compared to that in the non-treated group at 20 wps (Fig. 5c). However, paradoxically, its expression was upregulated at 40 wps in the metformin-treated group (Fig. 5c). IFN-γ levels in M1Ms at 40 wps were slightly but significantly decreased upon metformin treatment (Fig. 5c). In M2Ms, the expression of TGF-β and IL-10 was increased at 40 wps (carcinogenesis phase) (Fig. 5d). These increases were downregulated upon metformin treatment, suggesting that metformin stimulated immunological modulations (Fig. 5d). These findings led us to investigate macrophage p-Stat3 (pTyr705 and pSer727) levels. We could not observe any significant differences in p-Stat3 (pTyr705) levels among the groups (Fig. 5e). The p-Stat3 (pSer727) expression patterns in the non-treated control mirrored those of TNF-α, consistent with the percentage of M1Ms and similar to the expression patterns of TGF-β, IL-10, and the percentage of M2Ms (Fig. 5c–e). Metformin treatment inhibited the upregulation of macrophage p-Stat3 (Ser727) levels at 40 wps (Fig. 5e).

## Discussion

Chronic inflammation caused by extrinsic (infection, autoimmune disease, or caustic chemical injury) and intrinsic (genetic alterations) factors is a risk factor for carcinogenesis of the gastrointestinal tract, including EC [22]. In our longstanding rat surgical model, which is triggered by exposure to bile acid and chronic inflammation, EC develops spontaneously due to chronic GERD [13]. This spontaneous carcinogenesis model has morphological and pathological features similar to human clinical EC. The present macro- and microscopic examinations revealed the development of BE in almost all animals at 20 wps, followed by EC in 67% of the experimental animals at 30–40 wps (Fig. 3b). This occurrence rate was almost the same as the rate was reported previously [23].

**Fig. 6:**
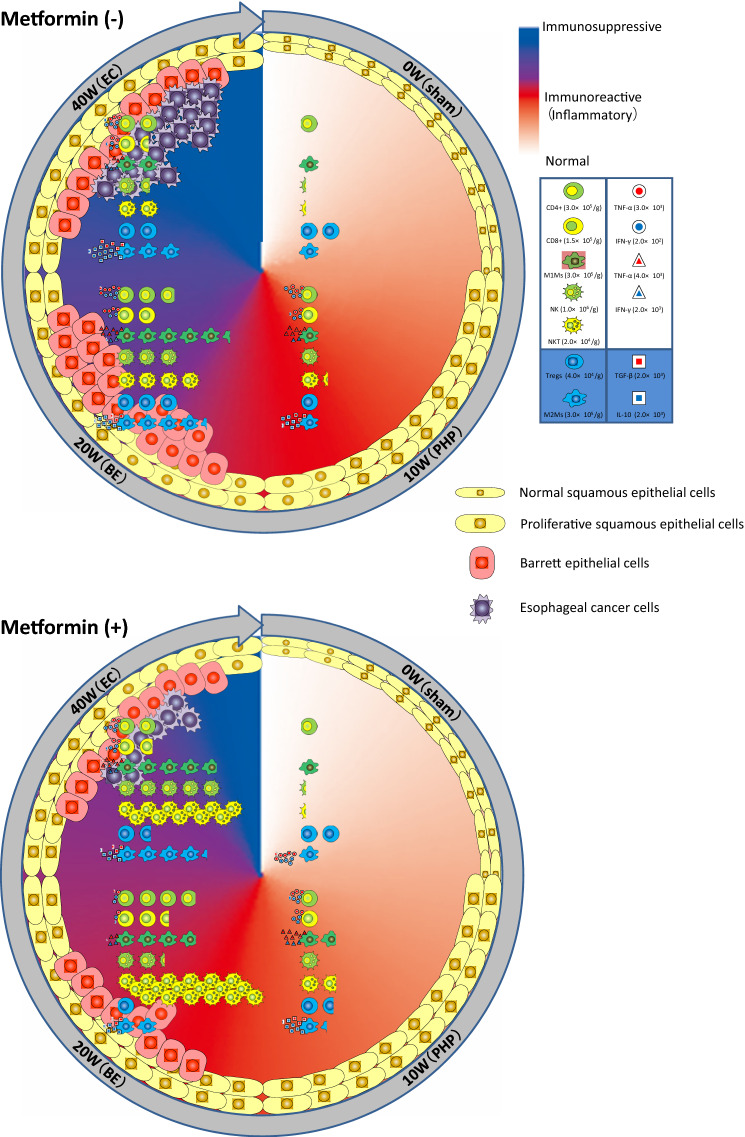
Illustrated here are dynamic changes of immune cell populations and characteristics during the carcinogenic transition from gastroesophageal reflux disease (GERD) to Barrett’s esophagus (BE) and finally to esophageal cancer (EC) in the esophagus of this rat model. Inflammatory reaction is initiated around 10 weeks after the surgery and proliferative hyperplasia (PHP) of the esophagus epithelial cells is observed. Metformin could impact the modulation of pro-inflammatory reactions in esophageal carcinogenesis and host antitumor immunity by improving the immunosuppressive tumor microenvironment and immune evasion

Cancer cells stimulate and promote the formation of a tumor microenvironment that can suppress antitumor immunity and obtain the nutrients for their growth and progression, in the same manner as in esophageal carcinogenesis [24]. The inhibition of antitumor immunity typically includes an increase in M2Ms and Tregs, accompanied by a decrease in M1Ms, CD8^+^ T, NK, and NKT cells surrounding the tumors [25, 26]. Previous studies have investigated the difference in the immune composition between normal esophagus, reflux esophagitis, BE, and EC using human biopsies or mouse models [27–29]. In addition to the analyses of the dynamic changes in the immune composition of chronic inflammation on reflux esophagitis and the tumor microenvironment of EC using the rat surgical carcinogenesis model, we examined the immunomodulating effect of metformin, which shows inhibitory activity against STAT3. Our overall findings across all phases of esophageal carcinogenesis and the dynamic changes in the populations and characteristics of immune cells with or without metformin treatment in this model are summarized in Fig. 6.

Under baseline natural conditions, CD4^+^ T cells, NK cells, and both M1Ms and M2Ms were detectable in the esophagus (Fig. 2). In particular, the immunosuppressive Treg cell population was predominant (Fig. 4). CD8^+^ T and NKT cells were rarely observed (Fig. 2). Tissue resident Treg cells and macrophages are essential for gut homeostasis because they regulate the mucosal immune response [30, 31]. After the onset of reflux esophagitis, both pro- and anti-inflammatory cells were recruited to the esophagus. The former included CD4^+^ T, Th17, CD8^+^ T, M1Ms, NK, and NKT cells. The latter included Treg and M2M cells. These inflammatory reactions were strongly promoted and peaked at 20 wps in esophagitis (Barrett rising phase). During this phase, the NK cell population was markedly increased and TNF-α levels in CD4^+^ and CD8^+^ T cells were upregulated (Fig. 4l, i). At 40 wps, during the carcinogenesis phase, the proportions of tumor-suppressive cells that included M1M, NK, and NKT cells were reduced, while those of tumor-promoting cells that included M2Ms and Treg cells were less affected (Figs. 4, 5). However, from a functional perspective, a significant increase in the ratio of TGF-β and IL-10 positive cells in Treg cells, the marked elevation of the expression of TGF-β and IL-10 in M2Ms, and the notably higher phosphorylation of STAT3 (Ser727) in macrophages was noted (Figs. 4, 5). A previous report described that IL-10-mediated activation of STAT3 promoted M2-polarization in macrophages [32]. These findings demonstrated that the tumor microenvironment shifts from a more immune-suppressive or immune evasion in the period from the Barrett rising phase to the carcinogenesis phase [25].

In terms of the effects of metformin, the populations of both pro- and anti-inflammatory cells were increased in the reflux esophagitis phase compared to those in the untreated control (Figs. 4, 5). At 20 wps, the NKT cell population was expanded and, in contrast, Treg cell expansion was largely inhibited and the levels of immunosuppressive cytokines were decreased (Fig. 4). The findings are compatible with previous reports showing that metformin inhibited Treg cell differentiation and function by reducing FoxP3 expression [33, 34]. Furthermore, metformin downregulated the expression of TNF-α in CD8^+^ T and M1M cells (Figs. 4, 5). The immune cell contents and patterns in untreated controls at 20 wps closely resembled those in the metformin-treated esophagus samples at 40 wps, suggesting that metformin treatment delayed the development of esophageal carcinogenesis as a chemopreventive drug.

In summary, we provide the first evidence of dynamic changes in the infiltration of immune cells during the carcinogenic transition from GERD to BM and finally to EC in the esophagus. The results show that immunomodulating agents, such as metformin, could potentially serve as preventive and therapeutic strategies against esophageal carcinogenesis.
